# Analogue Orientation Control of a Carbon Fibre in a Nematic Liquid Crystal

**DOI:** 10.1038/s41598-019-56594-6

**Published:** 2019-12-27

**Authors:** Jun-Yong Lee, Bohdan Lev, Jong-Hyun Kim

**Affiliations:** 1 Department of Physics, Chungnam National University, 99 Daehak-ro, Yuseong-gu, Daejeon 34134 Korea; 2 Bogolyubov Institute for Theoretical Physics of the NAS of Ukraine, Metrolohichna Str.14-b, Kiev, 03680 Ukraine

**Keywords:** Fluidics, Liquid crystals

## Abstract

A carbon fibre is a rod-like microstructure, the longitudinal axis of which is aligned with the orientation of the director in a nematic liquid crystal. A nematic liquid crystal with negative dielectric anisotropy is mixed with carbon fibres. By applying an electric field perpendicular to the director, the carbon fibres tend to rotate in response to the electric field, and the directors around the carbon fibres tends to suppress the rotation. We control individual carbon fibres to obtain an expected orientation by handling the competition of two actions. The carbon fibre barely reacts in a small electric field. Meanwhile, when the threshold electric field is exceeded, the carbon fibre rotates with a steep gradient in the direction of the electric field. The change in the rotation shows little hysteresis. As the length of the carbon fibre is increased, the threshold electric field decreases. We analysed the above process with a theoretical model considering the response of the carbon fibre and liquid crystal. This study shows the possibility of accurate analogue orientation control of individual rod-like microstructures.

## Introduction

A carbon fibre (CF) is a type of carbon compound and has a rod-like shape. It is used in a variety of industries, including in composites, fibres, and electrodes, owing to its excellent physical properties, such as high tensile strength, low coefficient of thermal expansion, high electric conductivity, and low density^1^. A CF consists of a stacked graphitic carbon layer similar to the graphite of a 2-dimensional material. Carbon atoms are formed by covalent bonding in the x-y plane, and the layers are formed by van der Waals bonding. Graphite shows anisotropic properties, such as the electrical and thermal conductivity, between the directions parallel and perpendicular to the layers. A CF also has anisotropic properties. For example, a CF is a good conductor in the direction parallel to the fibre axis, but it is not a relatively good conductor in the direction perpendicular to the fibre axis. Carbon nanotubes (CNTs), which are widely used as other carbon compounds due to their excellent physical properties, also exhibit physical and electric anisotropy. In general, we can enhance the anisotropic properties by aligning the rod-like particles in a uniform direction. Many studies have been conducted on aligning rod-like particles, especially CNTs, to improve their mechanical and electrical properties. The degree of the alignment was investigated with optical microscopy or Raman spectroscopy after applying an external electric field, measuring the order parameter of CNTs by applying an AC field to an aqueous suspension^2–8^. Furthermore, many studies have also been conducted on the liquid crystalline phase induced by rod-like particles^9–11^.

A liquid crystal (LC) is appropriate for studying the particles exhibiting anisotropies. An LC is a material with an intermediate phase between a crystal phase and the liquid phase^12,13^. Amongst the many LC phases, the nematic phase, which has the orientational order of a crystal phase and the liquidity of a liquid phase, is most widely used. An LC also has anisotropic properties, such as the dielectric constant and refractive indices, due to the orientational ordering. If the LC directors are not uniformly aligned, then the free energy of the system rises and becomes unstable. Furthermore, the behaviour of the LC directors at the interface is determined by the interaction with the boundary, which affects the behaviour of the directors in the bulk^14^. When one puts a colloidal particle into an LC, the LC directors around the particle deform due to the interaction with the particle. The deformation is determined by the shape, size, and surface condition of the particle^15,16^.

There are many studies on the interaction between LCs and rod-like particles, such as nanorods, microrods, and CNTs^17–26^. Rod-like particles can be controlled by an LC. The orientation of the rod-like particles in a uniformly aligned LC cell is determined by the surface condition. Normal anchoring makes the particles align perpendicular to the alignment orientation of the LC, and planar anchoring makes the particles align parallel to the alignment orientation. We can easily align rod-like particles, such as CNTs, due to the alignment property of LCs. When one changes the orientation of LC directors by applying an electric field, the orientation of the particles in the LC cell is also changed. Furthermore, the particles in LC cells change the properties of the LC. For example, CNTs distributed at an appropriate concentration in a LC cell reduce the ion concentration, lowering the driving voltage and dramatically decreasing the electrical conductivity^27–33^. In addition, CNTs in an LC affect many other properties, such as the LC cell viscosity, response time, dielectric constant, and phase transition temperature; thus, they can improve the performance of an LC device. In the existing studies, the collective average response of a large number of CNTs has been observed.

In this study, we investigated the static and dynamic response of individual CFs to an electric field, going beyond the existing studies of handling numerous rod-like particles on average.

## Experiments and Results

We used *N*-(4-methoxybenzylidene)-4-butylaniline (MBBA from TCI) as a nematic LC. MBBA has a negative dielectric anisotropy. The CFs (from Nanoshel) are 3~5 ${\rm{\mu }}{\rm{m}}$ in radius, 20~140 ${\rm{\mu }}{\rm{m}}$ in length, and approximately 2 g/${{\rm{cm}}}^{3}$ in density. We made a mixture by putting a small amount of CFs into MBBA and vigorously shaking the mixture bottle. We observed isolated CFs that were positioned far from other CFs to measure the response of individual CFs. All the processes were conducted at room temperature because MBBA is in the nematic phase at room temperature.

**Figure 1:**
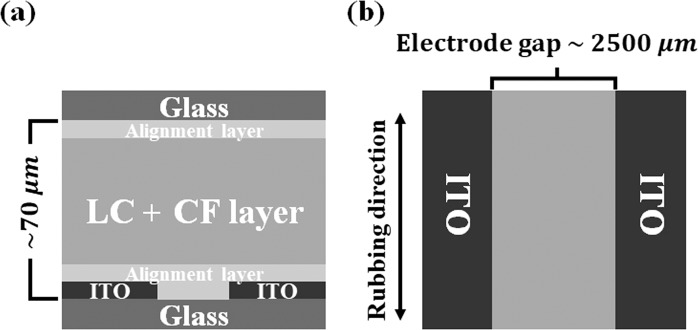
Schematic diagram of the LC cell: (**a**) side view and (**b**) top view on the bottom substrate. The relative size of each component in the diagram is adjusted for clarity of the cell structure.

We prepared LC cells as shown in Fig. 1. One side of the LC cell had a bare glass substrate, and the other side had an ITO glass substrate etched with an approximately 2500 µm electrode gap to be able to apply an electric field parallel to the substrate. Each substrate was spin-coated with a planar-aligning polyimide layer (PIA-PI114-01X from JNC), cured, and rubbed perpendicular to the electrode. Both substrates were fixed with double-sided tape with a thickness of approximately 70 ${\rm{\mu }}{\rm{m}}$. The MBBA and CF mixture was then injected into the cell.

A 5 kHz square wave electric field was applied with a function generator and an amplifier. The responses of the CF and texture were observed and analysed with a polarizing optical microscope.

Observation of the CF response proceeded in two ways. First, after applying an electric field of a certain strength, we waited for a sufficient time for the particular CF to reach equilibrium and then observed the orientation of the CF. We repeated this process by changing the strength of the electric field. Second, by applying a strong enough electric field to the ground state cell, we observed the rotation process until the CF reached the equilibrium state. Then, upon reaching the equilibrium state and turning off the electric field, we also observed the relaxation of the CF to the ground state. We analysed and measured the orientation change.

**Figure 2:**
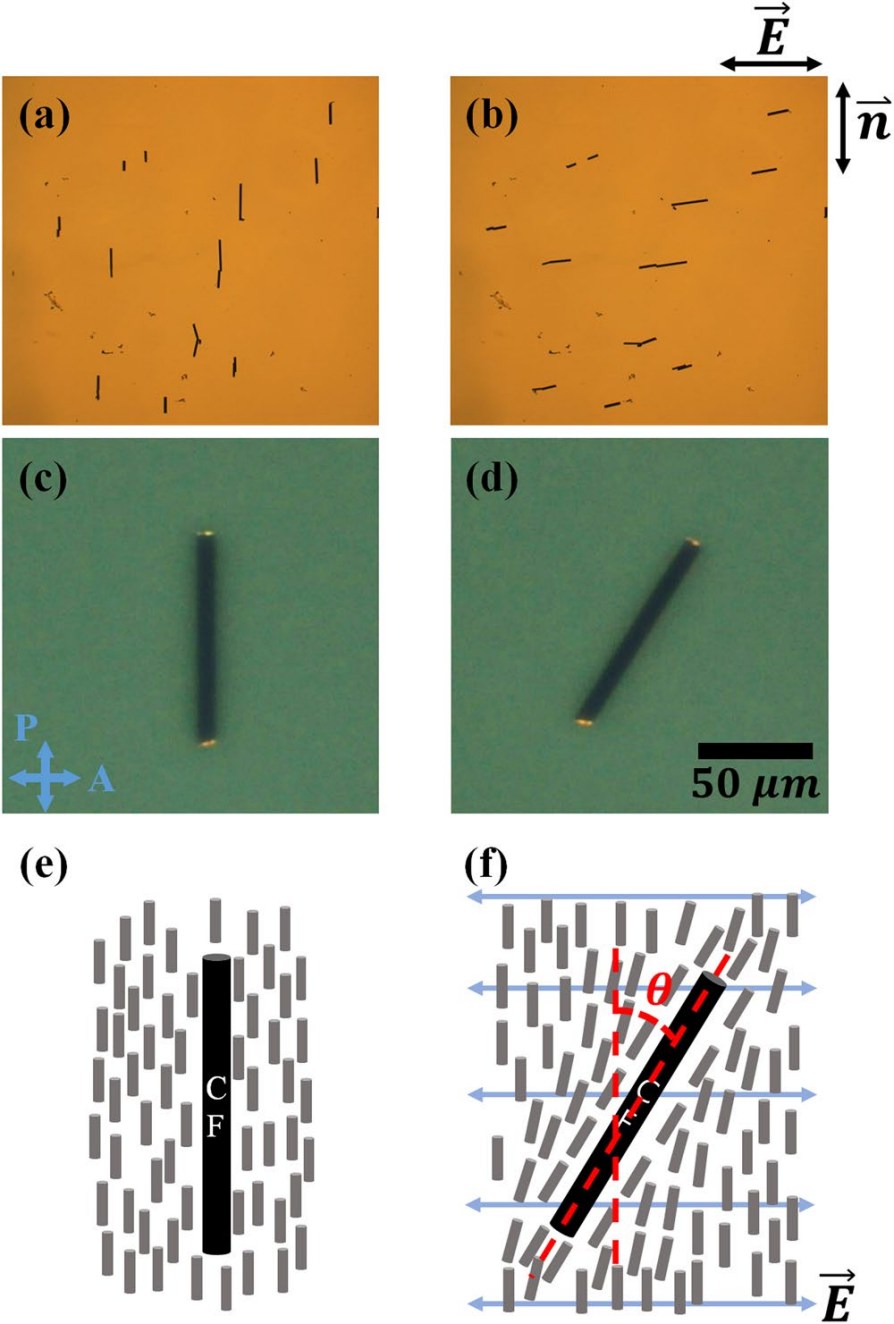
(**a**) Texture in the ground state of CFs obtained with an optical microscope. The tiny black lines are CFs. (**b**) Texture when an electric field is applied. (**c**) Texture in the ground state of a CF obtained with a polarizing optical microscope. (D) Texture when an electric field is applied. We adjusted the overall brightness for clarity of the image. (**e**) Schematic diagram of a CF and surrounding LC directors when in the ground state and (**f**) An electric field is applied. The black bar is the CF, and the small grey bars around the CF are directors. *θ* is the rotation angle of the CF.

It is known that on the surface of a CNT, the LC directors are aligned with the orientation parallel to the axis of the CNT^34^. The LC directors can be considered to be aligned with the fibre axis of the CF similar to in the case of a CNT. Therefore, if there is no external field, then the fibre axis of the CF is aligned with the orientation parallel to the LC directors. We confirmed this with the texture in Fig. 2. We can reorient the CF with a sufficient electric field due to the electric anisotropy, in which an electric dipole moment is generated mainly parallel to the fibre axis^35^. If the LC has a positive dielectric anisotropy, then the directors and the CF are aligned in the same orientation under the electric field, so the two effects may be mixed and disturb the analysis. To avoid this problem, we used an LC of negative dielectric anisotropy.

When an electric field is applied, the electric free energy due to the dipole moment of the CF, the deformation and the electric free energy of the LC play important roles. The CF and the LC directors rotate until reaching the orientation that minimizes the energy of the system. However, because the electric field is applied perpendicular to the bulk LC director, there is no rotation of the bulk LC under the electric field, and only the directors around the CF deviate from the field direction Fig. 2(e,f). We assumed that the anchoring strength on the surface of the CF is very strong.

When one puts colloidal particles in or applies an external field to an LC system, deformation of the LC directors occurs^12,13^. One can express the deformation as the combination of splay, twist, and bend deformation^12,36^. The free energy density (f) due to the elastic deformation is as follows, where *K*_1_, *K*_2_, and *K*_3_ represent the elastic constants of splay, twist, and bend deformation, respectively.

1 $${\rm{f}}=\frac{1}{2}[{K}_{1}{(\nabla \cdot \mathop{n}\limits^{\rightharpoonup })}^{2}+{K}_{2}{(\mathop{n}\limits^{\rightharpoonup }\cdot \nabla \times \mathop{n}\limits^{\rightharpoonup })}^{2}+{K}_{3}{(\mathop{n}\limits^{\rightharpoonup }\times \nabla \times \mathop{n}\limits^{\rightharpoonup })}^{2}]\,$$

$\mathop{n}\limits^{\rightharpoonup }$ is the director vector with unit magnitude. With a one-constant approximation, Eq. (1) can be simplified as follows.

2 $${\rm{f}}=\frac{1}{2}K[{(\nabla \cdot \mathop{n}\limits^{\rightharpoonup })}^{2}+{(\nabla \times \mathop{n}\limits^{\rightharpoonup })}^{2}]\,$$

Consider a cylindrical CF with length *L* and radius *r* lying inside a uniformly aligned LC cell, as shown in Fig. 2; the orientation of the CF changes when an electric field is applied. Considering the dipole moment (*p*) of the cylinder when the electric field is applied to the cylindrical conductor parallel to the axial direction, the dipole moment is expressed as follows^35^.

3 $$p={C}_{1}\cdot \frac{4{\rm{\pi }}{\epsilon }_{0}\kappa {L}^{3}}{24[lna-1]}[1+\frac{4/3-ln2}{lna-1}]\cdot E\,$$

C_1_~2/5 is a coefficient used to compensate for the deviations from the ideal conductor. *E* is the strength of the electric field, and *a* is *L*/*r*. *κ*~4.9 is the mean value of the relative dielectric constant of the LC^37^. ${\epsilon }_{0}$ is the electric permittivity in vacuum. Because the anisotropy of the CF is very large, we neglected the dipole moment perpendicular to the fibre axis. After applying the electric field, as shown in Fig. 2(d,f), we analysed the CF rotation, where the change has been expressed as the angle between the rubbing direction and the fibre axis (*θ*). Then, we can express the component of the electric field parallel to the fibre axis as *E* sin*θ*. The torque on the CF (${\Gamma }_{elec}$) and the electric free energy of the CF (${{\rm{F}}}_{{{\rm{CF}}}_{{\rm{elec}}}}$) are as follows.

4 $${\Gamma }_{elec}=p\times E=\frac{2}{5}\frac{4\pi {{\epsilon }}_{0}\kappa {L}^{3}}{24[lna-1]}[1+\frac{4/3-ln2}{lna-1}]\cdot Esin\theta \cdot Ecos\theta \,$$

5 $${{\rm{F}}}_{{{\rm{CF}}}_{{\rm{elec}}}}=-{\int }^{}{\Gamma }_{elec}d\theta =-\frac{1}{5}\frac{4\pi {{\epsilon }}_{0}\kappa {L}^{3}}{24[lna-1]}[1+\frac{4/3-ln2}{lna-1}]\cdot {{\rm{E}}}^{2}\,{\sin }^{2}\,\theta $$

When the CF orientation changes due to the electric field, LC deformation occurs as shown in Fig. 2(f). Assuming that the anchoring is very strong on the surface of the CF, we neglected the director deviation on the surface. If the region surrounding the cylinder is infinite with the ideal infinite length, then the distortion surrounding the cylinder affects the LC far from the cylinder. However, we can consider the region distorted by the cylinder as being on the order of the diameter of the cylinder due to the constrained cylinder length, electric coherence length, distance to the near substrate and so on. As the distortion is inversely proportional to the region of the distortion, the elastic deformation energy is not sensitive to the radius of the CF. Therefore, we can approximate the elastic deformation energy (${{\rm{F}}}_{{\rm{deform}}}$) as follows^38^.

6 $${{\rm{F}}}_{{\rm{deform}}}=\frac{\pi KL}{\mathrm{ln}(a/2)\,}{\theta }^{2}\,$$

With the approach used above, the electric free energy (${{\rm{F}}}_{{{\rm{LC}}}_{{\rm{elec}}}})$ can be expressed as follows.

7 $${{\rm{F}}}_{{{\rm{LC}}}_{{\rm{elec}}}}={f}_{L{C}_{elec}}\times {V}_{deform}=-\,\frac{1}{2}{\epsilon }_{0}\Delta {\epsilon }_{{\rm{MBBA}}}{{\rm{E}}}^{2}{\sin }^{2}\frac{\theta }{2}\times 8{\rm{\pi }}{r}^{2}L\,$$

In the above equation, ${f}_{L{C}_{elec}}$ is the electric energy density of the LC and ${V}_{deform}$ is the effective volume of the LC, which are related to the electric field, and $\Delta {\epsilon }_{{\rm{MBBA}}}$ is the relative dielectric anisotropy of the LC. Assuming that the director varies from the surface of the CF to a distance on the order of the diameter, the orientation of the director is taken as the mean value for simplicity. Given that the dielectric anisotropy of the LC used in the experiment is very small, ${{\rm{F}}}_{{{\rm{LC}}}_{{\rm{elec}}}}$ (Eq. (7)) is relatively small compared to ${{\rm{F}}}_{{{\rm{CF}}}_{{\rm{elec}}}}\,$(Eq. (5)) and ${{\rm{F}}}_{{\rm{deform}}}$ (Eq. (6)). Therefore, the effect of ${{\rm{F}}}_{{{\rm{LC}}}_{{\rm{elec}}}}$ on the behaviour of the CF under an electric field is small such that it barely affects the simple approximation calculation.

We used 7.3 pN as K in Eq. (6), which is the mean value of the elastic constants of MBBA^39^. $\Delta {\epsilon }_{{\rm{MBBA}}}$ in Eq. (7) is −0.62^37^. For the static case, upon increasing the electric field step-by-step, we calculated the total free energy of the system considering Eqs. (5), (6), and (7). We considered only the terms with $\theta$ dependence because we deal only with the rotation of the CF.

8 $${{\rm{F}}}_{{\rm{total}}}=-\,\frac{1}{5}\frac{4\pi {{\epsilon }}_{0}\kappa {L}^{3}}{24[lna-1]}[1+\frac{4/3-ln2}{lna-1}]\cdot {{\rm{E}}}^{2}{\sin }^{2}\theta +\frac{\pi KL}{\mathrm{ln}(a/2)}{\theta }^{2}-\,\frac{1}{2}{\epsilon }_{0}\Delta {\epsilon }_{{\rm{MBBA}}}{{\rm{E}}}^{2}{\sin }^{2}\frac{\theta }{2}\times 8{\rm{\pi }}{r}^{2}L\,$$

We expected that the static equilibrium state is reached at the angle $\theta$ that satisfies ${{\rm{dF}}}_{{\rm{total}}}/{\rm{d}}{\rm{\theta }}=0$. Further, to calculate the dynamics of the process of reaching equilibrium, we must consider the torque on the CF, including the viscosity effect of the LC layer. With the existing studies on CNTs, we can calculate the rotational friction coefficient as follows^40,41^.

9 $${\Gamma }_{vis}={{\rm{\zeta }}}_{{\rm{R}}}\frac{d\theta }{dt}\,=\frac{\pi {\eta }_{s}{L}^{3}}{3[lna-\beta ]}\frac{{\rm{d}}{\rm{\theta }}}{{\rm{dt}}}\,$$

${\Gamma }_{vis}$ is the viscous torque and ${{\rm{\zeta }}}_{{\rm{R}}}$ is the viscous coefficient. ${\rm{\beta }}$ is a geometric correction factor and has a value of 0.8 for a cylinder. Within an LC, the viscosity varies depending on the LC alignment orientation, and the viscosity also varies with the angle and position upon CF rotation. Considering this, we used 91.5 ${\rm{mPa}}\cdot {\rm{s}}$ as ${{\rm{\eta }}}_{{\rm{s}}}$, which is the mean value of the viscous coefficient of MBBA^42^. The differential of the free energy of the LC in Eqs. (6) and (7) acts as a torque induced by the LC layer, and we obtain the following equations.

10 $${\rm{I}}\frac{{{\rm{d}}}^{2}\theta }{d{t}^{2}}={\Gamma }_{elec}-\frac{d{F}_{deform}}{d\theta }-\frac{d{F}_{L{C}_{elec}}}{d\theta }-{\Gamma }_{vis}\,$$

11 $${\rm{I}}\frac{{{\rm{d}}}^{2}\theta }{d{t}^{2}}=-\,\frac{d{F}_{deform}}{d\theta }-{\Gamma }_{{\rm{vis}}}\,$$

I is the moment of inertia of the CF. Equations (10) and (11) indicate the response when an electric field is applied and the relaxation when the electric field is turned off, respectively. Because the value of I is very small compared to the other terms, we ignored ${{\rm{Id}}}^{2}\theta /d{t}^{2}$ in the calculation.

**Figure 3:**
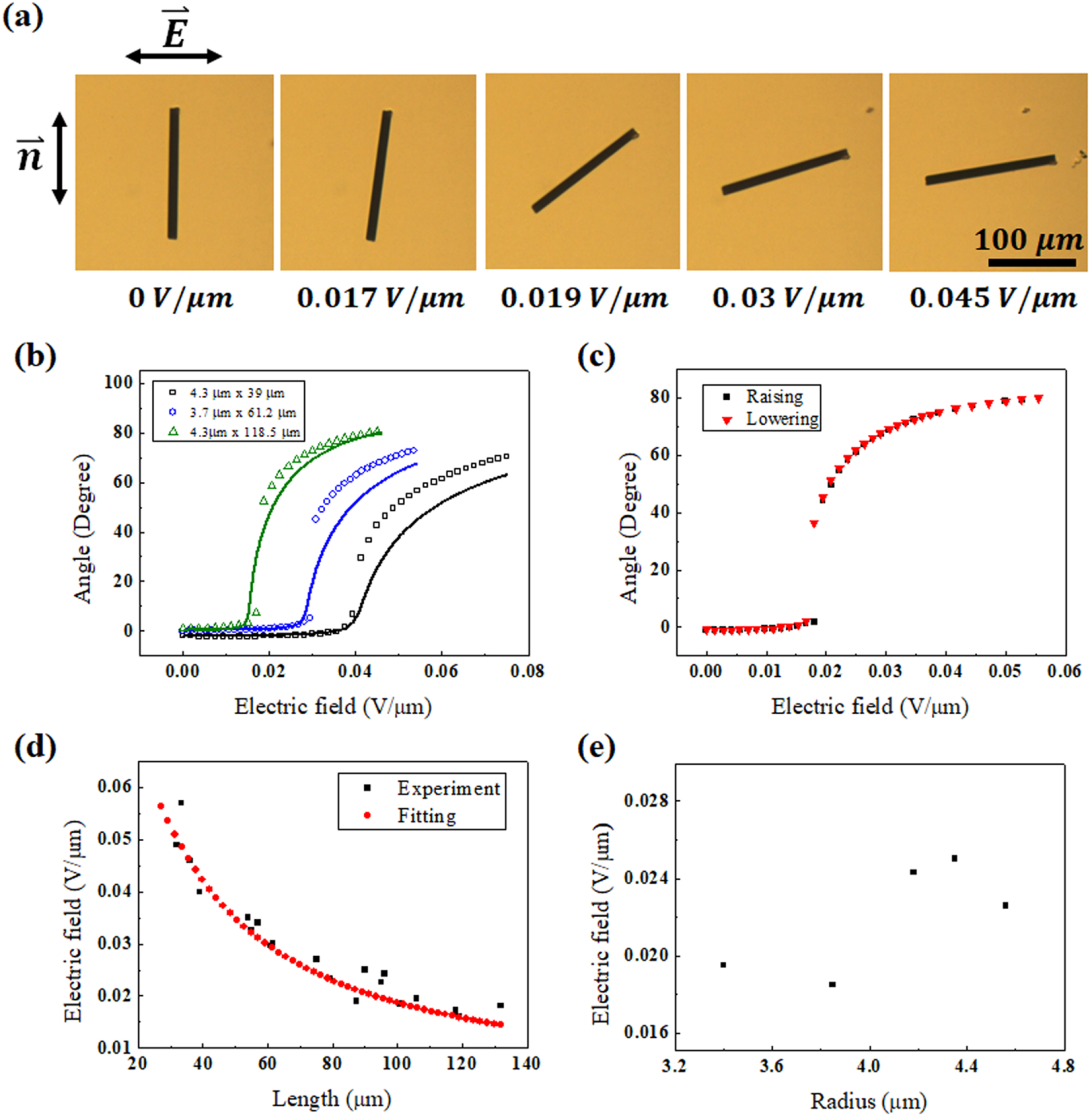
(**a**) Images of a CF for different electric fields. (**b**) Graph of the experimental and fitting results of (**a**), calculated with Eq. (8). Empty squares are experimental results, and solid lines are the calculations. (**c**) Graph of the rotation angle of a CF with increasing and decreasing electric field strength. (**d**) Graph for comparing the experimental results with the calculation of the threshold electric field as a function of CF length. (**e**) Threshold electric field for changing radius of CFs with similar length (90~106 µm).

Applying an electric field perpendicular to the director and gradually changing the strength, we observed the orientation change of a CF with the optical microscope, as shown in Fig. 3(a). In the small electric field range, the CF barely responds; however, beyond the threshold electric field, the CF drastically responds. This is analogous to the first-order phase transition or Frederick transition [Fig. 3(b)]. The response shows a clear trend with the CF length. The angle change of a longer CF starts at a smaller electric field and saturates faster compared to the angle change of a shorter CF. There is little hysteresis between the increasing and decreasing field change directions, as shown in Fig. 3(c). We repeated the measurement cycling of the electric field with the same CFs, and there was little difference between cycles. When the cell was heated to the isotropic phase, the controllability of the CF orientation was lost. In the entire range of electric field that can rotate the CF, the final rotation orientation was always parallel to the electric field even though the rotation speed was dependent on the electric field strength. Moreover, the CF does not return to the original orientation but stays at the rotated orientation.

We numerically calculated $\theta$ by minimizing Eq. (8) for CFs with different lengths and compared it with the experimental results. In the calculation, we considered that the fibre axis does not perfectly coincide with the rubbing direction and that the homemade electrode might not be perfectly perpendicular to the rubbing direction. However, there are still some differences between the experimental results and model calculations. In the region over the threshold electric field, the change in the rotation angle is more dramatic in the experimental results than in the calculation results. Nevertheless, it seems that the overall tendency of our model behaviour is appropriate.

Whereas the electric free energy contributing to the CF rotation is proportional to ${L}^{3}$, the deformation energy hindering it is proportional to ${L}^{2}$. Thus, we expected that the longer the CF was, the smaller the threshold electric field. In actuality, by analysing CFs with different lengths, the threshold electric field was indeed smaller for a longer CF, and we compared it to the theoretical calculation results, as shown in Fig. 3(d). We selected CFs of 90 ~106 µm in length and drew the threshold electric field as a function of radius as in Fig. 3(e). The threshold seems to be smoothly dependent on the radius. In the experimental data, we considered the median value of the steepest step as the threshold electric field. Under or near the threshold electric field, we deal with very small *θ*. Thus, we can approximate *θ* as near zero in Eq. (8). To simplify each term excluding *K*, *E*, and *θ*, we expressed the LC deformation, electric free energy of the CF, and electric free energy of the LC as ${\rm{\alpha }}$, ${\rm{\beta }}$, and ${\rm{\gamma }}$, respectively.

12 $$\begin{array}{c}{{\rm{F}}}_{{\rm{total}}}\cong [\frac{\pi KL}{\mathrm{ln}(a/2)}-(\frac{1}{5}\frac{4\pi {{\epsilon }}_{0}\kappa {L}^{3}}{24(lna-1)}(1+\frac{4/3-ln2}{lna-1})+{\epsilon }_{0}\Delta {\epsilon }_{{\rm{MBBA}}}{\rm{\pi }}{r}^{2}L){E}^{2}]{\theta }^{2}\\ \,\,=\,[K\alpha -{{\epsilon }}_{0}(\beta +\gamma ){E}^{2}]{\theta }^{2}\,\end{array}$$

Because the term for LC deformation energy is larger than the term for the electric field in the small electric field region, the CF is stable when *θ* is zero. However, with an electric field over ${E}_{c}=\sqrt{(K/{{\epsilon }}_{0})(\alpha /(\beta +\gamma )))}$, which is the threshold electric field, *θ* has a non-zero value. An electric field below *E*_*c*_ cannot rotate the CF, and *E*_*c*_ is the minimum electric field to rotate the CF. The equation enables us to obtain the *E*_*c*_ for the individual CFs of Fig. 3, such as 0.042 V/µm for 39 µm, 0.030 V/µm for 61.2 µm, and 0.016 V/µm for 118.5 µm. In the equation, when *a* =*L*/*r* is introduced, the behaviour becomes complicated by the changes in *L* and *r*. However, because $a$ is in a logarithmic function, the effect on the changes is small compared to the other factors, and the LC electric free energy contributes little to the transition. Considering these, *E*_*c*_ can be simplified as $\sqrt{(30K/{{\epsilon }}_{0}\kappa {L}^{2})}$ for a sufficiently large value of *L*/*r*, the length to radius ratio of the fibre, and *E*_*c*_ shows a trend of being inversely proportional to *L*, as shown in Fig. 3. However, the values at short lengths show different behaviours. The above *E*_*c*_ is shifted by the ratio $15(\Delta {\epsilon }_{{\rm{MBBA}}}/\kappa )\,lna({r}^{2}/{L}^{2})$ with the first approximation to *r*, and the maximum shift is approximately 5% within the variation of the length and radius of CFs used in the experiment. In terms of the LC, *E*_*c*_ is proportional to the square root of the elastic constant. This is the same behaviour as the response of the pure LC. The electric free energy of the LC prevents the CF from rotating due to the property of being aligned perpendicular to the electric field direction. As mentioned above, because $\Delta {\epsilon }_{{\rm{MBBA}}}$ is relatively small, the electric free energy of the LC is much smaller than that of the CF. In fact, for the CF 4 µm in radius and 60 µm in length, ${\rm{\beta }}\cong 8.74\times {10}^{-14}\,{m}^{3},\,|\gamma |\cong 1.87\times {10}^{-15}\,{m}^{3},\,{\rm{and}}|\gamma |/\beta \cong 0.02$. Thus, the effect of the electric free energy of the LC is very small.

**Figure 4:**
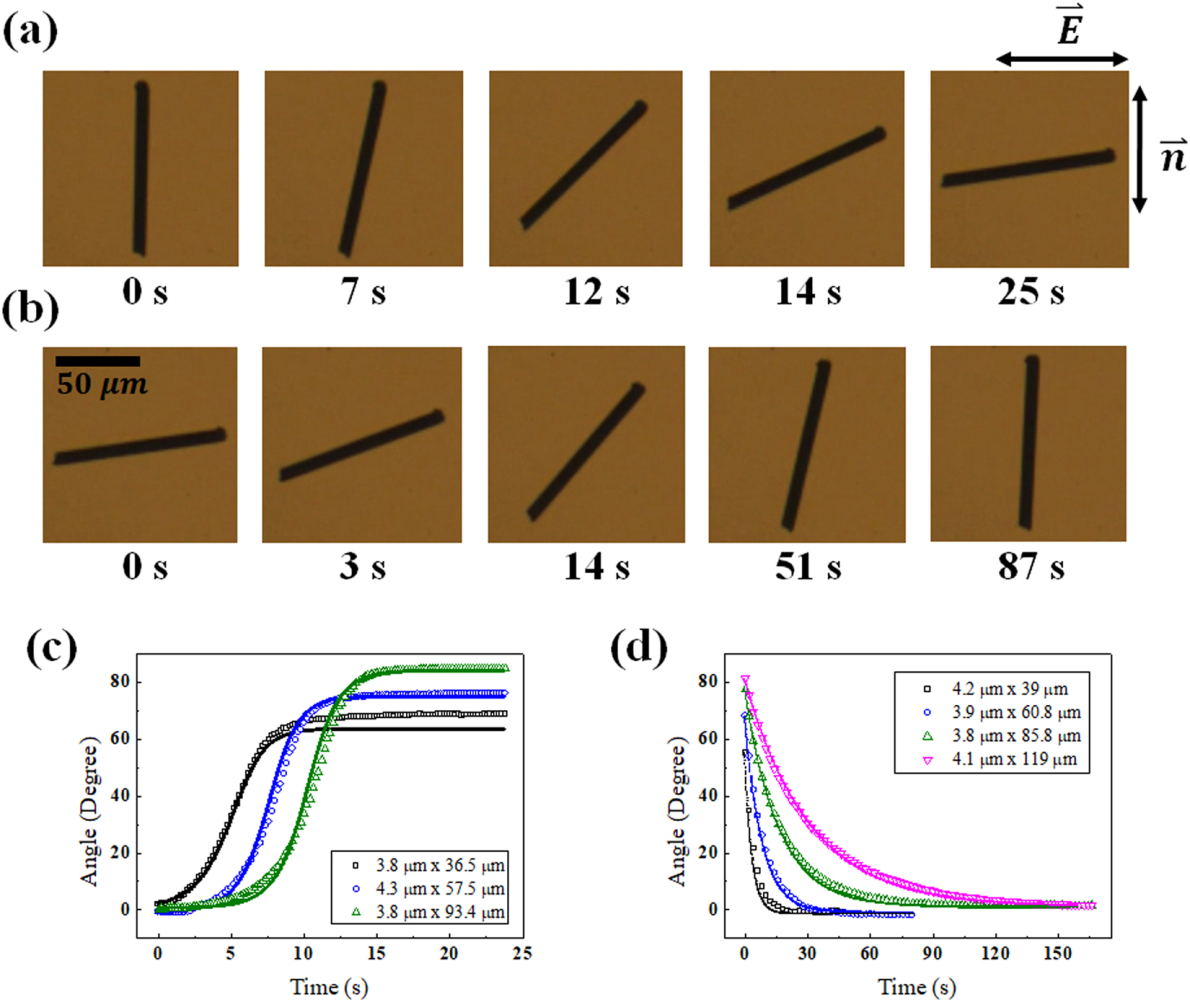
(**a**) Image of rotation of a CF according to the time elapsed when applying an electric field (0.075 V/µm). (**b**) Image of rotation by relaxation of the CF when the electric field is removed. (**c**) Graph of the process of (**a**) for different CFs. (**d**) Graph of the relaxation process of (**b**). The markers in (**c,d**) are the experimental results, and the lines indicate the model calculations for each experiment.

After applying an electric field (0.075 V/µm) sufficient for the CF to respond, we observed the process of the CF reaching equilibrium and relaxing to the ground state with the field off Fig. 4(a,b). The change of radius did not affect to the response and relaxation in comparison to the change of length. Analysing the angle change as time elapses, we plotted the graph and compared it to the results calculated from Eqs. (10) and (11) [Fig. 4(c,d)]. In the calculated result, we considered that the fibre axis might not perfectly coincide with the rubbing direction, and the homemade electrode might not be perpendicular to the rubbing direction. The on-time and off-time were defined as the elapsed time when going from an angle of 10% to 90% and from 90% to 10% of the saturation angle, respectively. We repeated the above experiment for CFs with different lengths and analysed the results. In the experiment to investigate the on/off-time for various length CFs, we used the electric field of 0.0525 V/µm for all the samples.

**Figure 5:**
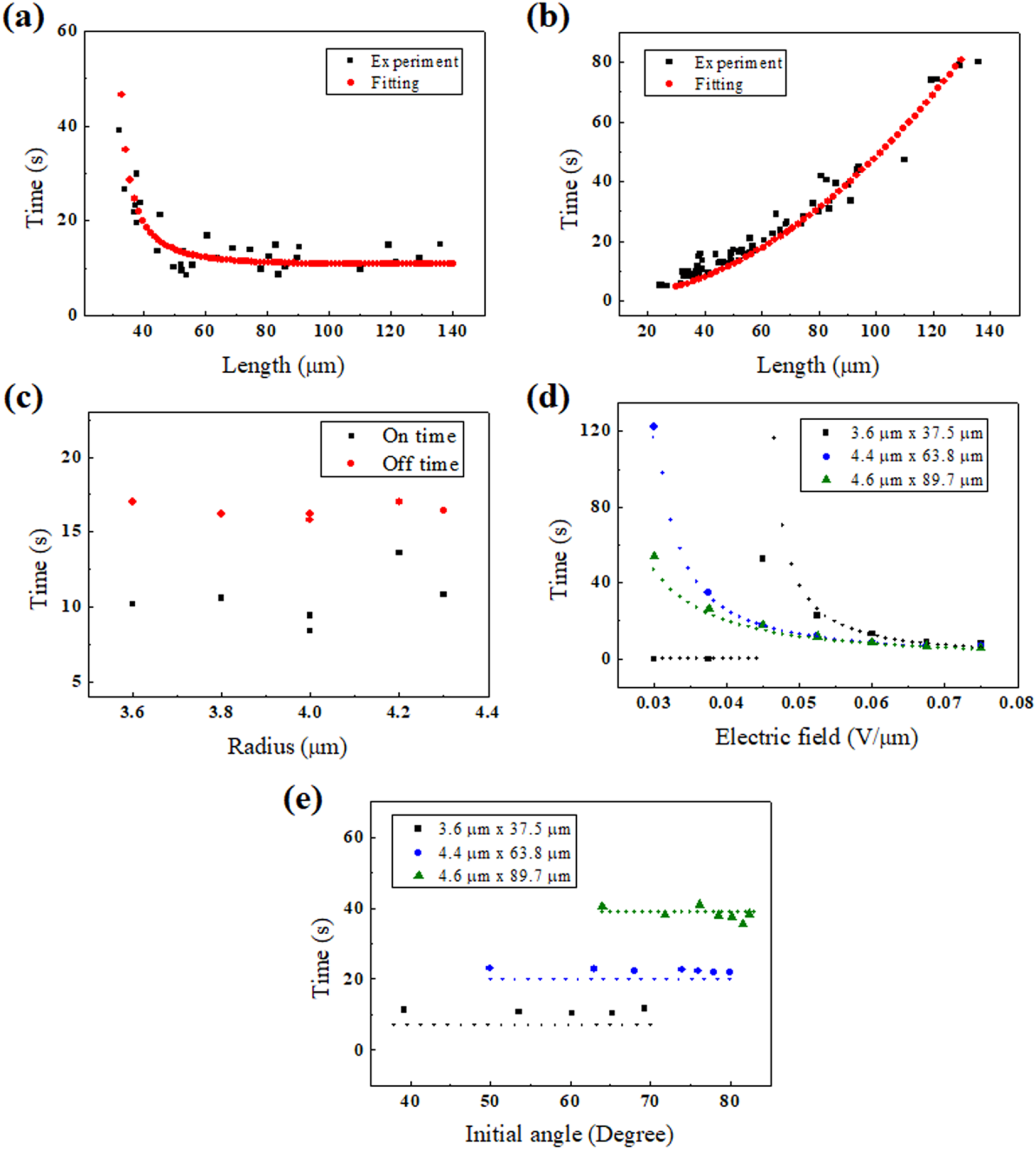
Experimental and calculation results of the (**a**) on-time as a function of CF length, (**b**) off-time as a function of CF length, (**c**) on- and off-time as a function of CF radius. The length of CF is in the range of 50~56 µm. (**d**) on-time as a function of the electric field for different CFs, and (**e**) off-time as a function of the initial angle for different CFs. The marks indicate the experimental results, and the small circles indicate the calculation results.

For longer lengths, the on-time is shorter. We numerically calculated the on-time as a function of length using Eq. (10) and compared it to experimental results of Fig. 5(a). From Eq. (11) related to the off-time, we obtained the following equation: $\theta (t)={\theta }_{0}\,\exp (-t/{\tau }_{off})$, ${\tau }_{off}=({C}_{2}/6)(\mathrm{ln}(L/2r)/(\mathrm{ln}(L/r)-0.8)\,{\eta }_{s}{L}^{2}$. We confirmed that the off-time is longer with longer CF, and the theoretical values of the time constant we obtained were compared with the experimental results of Fig. 5(b,c).

Furthermore, varying the strength of the electric field applied to the same CF, we analysed the response. When the electric field becomes stronger, ${\Gamma }_{{\rm{elec}}}$ contributing to the rotation of the CF becomes larger with an ${E}^{2}$ dependence, and the on-time becomes shorter Fig. 5(d). Although the initial angle is larger with a stronger electric field, as shown in Fig. 3(b), the off-time is constant because the time constant does not vary with the electric field for the same length in Fig. 5(e).

## Discussions

All CFs used in the experiments have a radius of 3~5 µm and a length of 20~140 µm. Given that the deviation in lengths is larger than that in radii, we analysed the experimental results for various lengths keeping the radius fixed (4 µm). In fact, the calculation results were not sensitive to the variation in radius in the limited range. Because the density is approximately 1 g/${{\rm{cm}}}^{3}$ for the LC and approximately 2 g/${{\rm{cm}}}^{3}$ for the CF, we might consider that the CF lies near the bottom substrate and not in the middle of the cell with a gap of 70 µm. However, we neglected the interaction with the substrate to simplify the analysis.

We obtain the deformation free energy as ${{\rm{F}}}_{{\rm{deform}}}={\rm{\pi }}KL{\theta }^{2}$ by assuming a linear deformation from the surface of the CF to a distance typically on the order of the diameter and neglecting the edge effect of the finite cylinder. Actually, there is a difference of ln(*L*/2*r*) from Eq. (6) that we used in the calculation. Thus, the value from Eq. (6) is smaller than that of the above free energy by a factor of ln(*L*/2*r*). Furthermore, Eq. (9) is the estimated calculation for a particle with a very high aspect ratio. The aspect ratio of the CF is somewhat different for each CF. Therefore, the effect of the edge or the change in ln(*L*/*r*) in the denominator is non-negligible.

**Figure 6:**
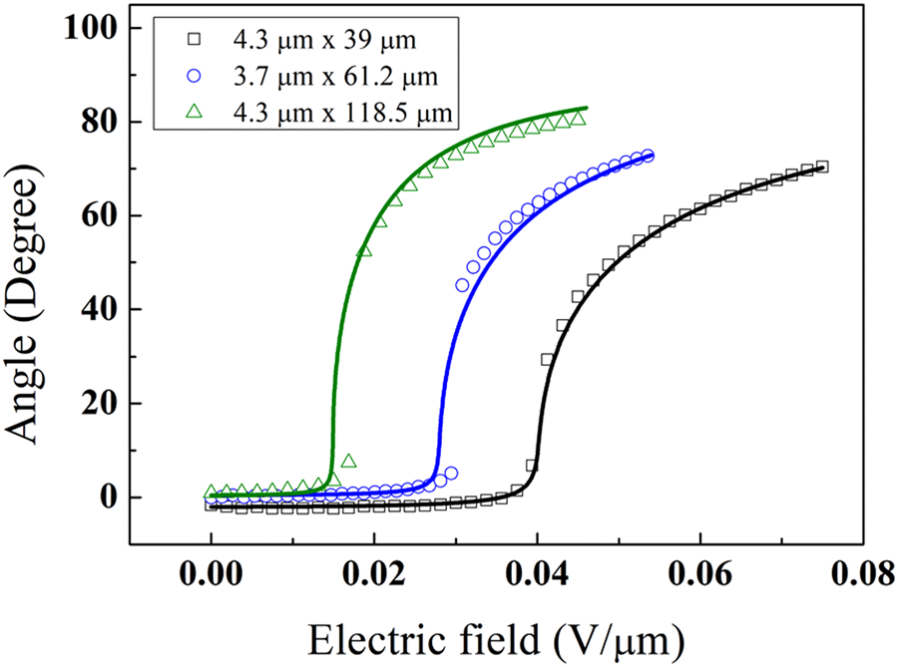
Comparison between experimental and calculated results. The calculation results are from the modified Eq. (5) with replacement of the sin^2^*θ* term by the (sin^2^*θ*  + 0.3 sin^3^*θ*) term. The hollow squares are the experimental results, and the solid lines are the calculation results.

As shown in Fig. 3(b), there is a difference between the experimental and calculation results in the rotation angle above *E*_*c*_; the change is more drastic in the experimental results. To improve the calculation, we tried to correct the ${{\rm{F}}}_{{{\rm{CF}}}_{{\rm{elec}}}}\,$of Eq. (5). Instead of the sin^2^*θ* term, we modified it as (sin^2^*θ* + 0.3sin^3^*θ*), adding the term 0.3 sin^3^*θ* . The modified calculation results match the experimental results very well, as shown in Fig. 6. In addition, adding a higher order of the sine function improves the fitting accuracy; however, it seems not critical. In general, it is assumed that the magnitude of the dipole moment is a constant that is a first approximation and appears to vary with orientation. Moreover, what is represented by this equation is that the nonlinear term of the electric field strength, which is a higher order approximation, is missing and that the nonlinear term of the orientation has entered. However, the specific reason is currently unclear.

We included the coefficient C_1_ in Eq. (3) for dealing with the real conducting properties deviating from those of a perfectly cylindrical conductor. Whereas Eq. (3) excluding C_1_ is the calculation for a perfect conductor, in fact, the CF might not be a perfect conductor with defects and the degree of alignment with the layers. We expected that the degree of generating the dipole moment for the imperfect conductor is smaller than the theoretical estimation. Considering these issues, we included the coefficient C_1_, whose value is 2/5, to make the calculation correspond to the experimental results.

When calculating Eq. (10), if we set the initial angle exactly to zero, then the CF does not respond when applying an electric field over the threshold. The reason is that the CF is in the equilibrium state at the angle *θ* that satisfies ${{\rm{dF}}}_{{\rm{total}}}/d\theta =0$, and the zero angle satisfies the unstable equilibrium state of the CF. In the actual experiment, it is difficult for the electrode to be perfectly perpendicular to the rubbing direction, and the initial angle of the CF might not coincide with the rubbing direction. Therefore, we observed no CF staying in the unstable equilibrium state at an electric field sufficient to rotate. To better understand the dynamics in the electric field, Eq. (10) can be expressed near $\theta =0$ simply as follows: ${\Gamma }_{vis}={\Gamma }_{elec}-d{F}_{deform}/d\theta -d{F}_{L{C}_{elec}}/d\theta$ and $D{L}^{3}({\rm{d}}\theta /{\rm{dt}})=A{L}^{3}{E}^{2}\theta -BL\theta -C{E}^{2}L\theta$. Here, *A*, *B*, *C*, and *D* represent the coefficients excluding *L* and *E* in each term. The solution is as follows: $\theta ={\theta }_{0}\exp \,(t/{\tau }_{on})\,$and ${\tau }_{on}=D{L}^{2}/((A{L}^{2}-C){E}^{2}-B)$. Because we address a wide range of rotation angles, the above equations cannot satisfy the detailed dynamics of the CF. However, using the solution obtained, we analysed the variation in the on-time with the CF length or the electric field strength. As previously mentioned, ${{\rm{F}}}_{{{\rm{LC}}}_{{\rm{elec}}}}$ is negligible, and we obtain a simple relation. The on-time variation with length converges to a particular value (∼D/*A*E^2^), whereas the on-time variation with the electric field is smaller with a stronger electric field. These tendencies correspond to the results of Figs. 5(a,d).

## Conclusions

The fibre axis of a CF is aligned parallel to the LC director in a uniformly aligned nematic LC. With a gradual increase in the electric field, the orientation of the fibre axis of the CF changes little in the low field strength range. The orientation starts to drastically change above *E*_*c*_ and smoothly saturates in the high field strength range. When the length of the CF is longer, *E*_*c*_ is lower and the equilibrium angle at a particular electric field is larger. We observed and analysed the dynamic processes for the CF to reach the equilibrium state by applying or removing an electric field, defining the on/off-time. The longer the length is, the shorter the on-time and the longer the off-time. We confirmed the possibility of controlling an individual rod-like shape, a CF, with the static and dynamic properties mentioned above. In addition, by appropriately controlling the strength of the electric field, we can reorient the CF to the desired orientation. We calculated all the processes with the theoretical model and compared them to the experimental results.

Existing studies of controlling a rod-like structure used a method to reorient LC directors to control the structure. Such a method has the problem that the effects of the LC and rod-like structures are mixed, which makes control and analysis difficult. In this study, an LC with a negative dielectric anisotropy was used to reorient the CF. In this method, the rotation of the CF is due only to the electric response of the CF without a change in the LC director. We expect that the results we obtained can help us better understand the electric and viscous properties of not only the CF but also other rod-like structures such as CNTs. Furthermore, we can not only control rod-like particles to any targeted orientation but also reversibly recover the orientation. We expect that this method will be used in microsized analogue switching devices.
